# The ultrastructural development and 3D reconstruction of the transparent carapace of the ostracod *Skogsbergia lerneri*

**DOI:** 10.1007/s00227-021-04006-7

**Published:** 2022-02-13

**Authors:** Benjamin M. Rumney, F. Tegwen Malik, Siân R. Morgan, Andrew R. Parker, Simon Holden, Julie Albon, Philip N. Lewis, Keith M. Meek

**Affiliations:** 1 School of Optometry and Vision Sciences, Cardiff University, Maindy Road, Cardiff, CF24 4HQ UK; 2 Swansea University, School of Management, Swansea, SA1 8EN UK; 3 Green, Templeton College, University of Oxford, Woodstock Road, Oxford, OX2 0HG UK; 4 DSTL Physical Sciences Group, Platform Systems Division, DSTL Porton Down, Salisbury, SP4 0JQ UK

**Keywords:** Ostracod, *Skogsbergia lerneri*, Serial block face scanning electron microscopy, Ultrastructure, Instar development, Transmission electron microscopy, Myodocopid, Volume electron microscopy

## Abstract

The *Skogsbergia lerneri* is a marine ostracod which possesses a carapace that is both protective and transparent. Since development of this carapace and how it is maintained in the adult is not known, the aim of this investigation was to carry out an in-depth ultrastructural study of the ostracod carapace at different developmental stages. Standard transmission electron microscopy and novel serial block face scanning electron microscopy (SBF-SEM) were undertaken to discern carapace ultrastructure in both two and three dimensions. Analysis revealed a carapace consisting of the same basic layer structure as other myodocopid ostracods, namely an epicuticle, exocuticle, endocuticle and membranous layer, but with a thinner adult carapace of mean thickness of 19.2 ± 1.78 µm, *n* = 5. The carapace layers, except for instar 1 ostracods, had similar relative proportions throughout development. The endocuticle and membranous layer thickened through advancing developmental stages due to an increase in calcified crystalline polyhedrons and a greater number of chitinous lamellae in the membranous layer. Crystalline polyhedron dimensions were significantly smaller near the boundary with the membranous layer. The borders between the carapace layers were indistinct; SBF-SEM revealed an abundance of epicuticle projections into the exocuticle and apparent gradual merging at the boundary of the exocuticle and the endocuticle. Here, we discuss how the *S. lerneri* carapace layer structure has evolved to serve a specific mechanical function, allowing surface protection and rigidity. In addition, we suggest that the lack of pigment and graduated layer boundaries contribute to the transparency of the carapace.

**Supplementary Information:**

The online version contains supplementary material available at 10.1007/s00227-021-04006-7.

## Introduction

Ostracods are small crustaceans (up to 32 mm in length) with a body that is entirely enclosed within a bi-valved carapace. The primary purpose of the carapace is protection from predators and physical environments. As such, the carapace is structured to provide the highest levels of protective mechanical properties possible. Ostracod carapaces, as in other crustaceans, generally consist of four main layers, a thin waxy outer coating known as an epicuticle, a chitinous exocuticle, an endocuticle and a membranous layer. The latter is composed of organised rows of chitin with differing layer densities (Stevenson 1985), which in this paper we refer to as lamellae. In most ostracods, the endocuticle contains a structure formed from calcium carbonate within an organic matrix (Vincent and Wegst 2004). Proximal to the carapace is a layer of epidermal cells that are responsible for the synthesis of the carapace. Pore canals traverse throughout the entire carapace; these carry out either sensory or mineral transfer roles (Maddocks 1990; Holmes and Chivas 2002) for the ostracod.

**Fig. 1:**
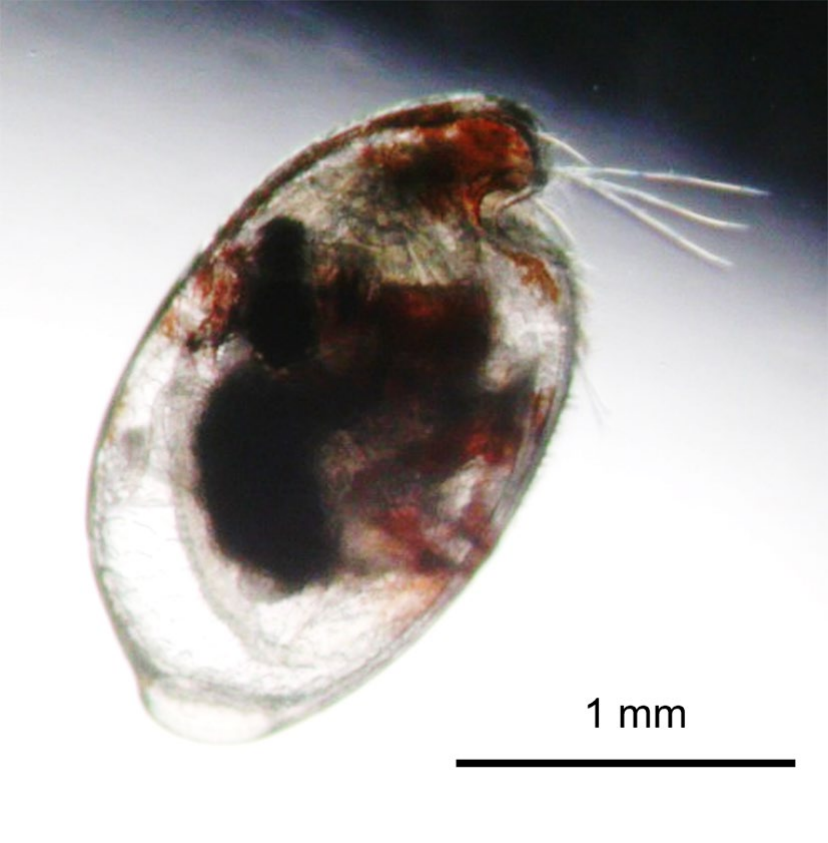
The ostracod *S. lerneri* possesses a highly transparent carapace through which the internal organs, including the eyes, are visible

This study focuses on the myodocopid ostracod family Cypridinidae, many of which use visual signals in the form of iridescence or bioluminescence for courtship (Parker 1995; Cohen and Morin 2003). The interest in these species is that their carapaces are not only protective, but appear to be highly transparent, at least in part, to allow their well-developed compound eyes to see. For example, our previous work (Parker et al. 2019) examined the ultrastructure of the carapace in *Macrocypridina castanea* (Brady, 1897), a large deep water cypridinid ostracod. These relatively large (around 10 mm in length) ostracods are brown in colour, with transparent windows in front of the eyes showing around 99% transmission of blue light. The membranous layer was found to occupy two-thirds of the carapace thickness, and transparency arises from lamellae that are very regularly spaced, forming a half-wave reflector. This permits optimum transmission of light in the visible range, particularly the blue and green wavelengths that best transmit in seawater (Parker et al. 2019). *Skogsbergia lerneri* (Kornicker 1958) is a small (about 2 mm) cypridinid ostracod that possesses a completely transparent carapace instead of the transparent eye window of *M. castanea* (Cohen 1983) (Fig. 1). However, in the case of *S. lerneri*, it is unknown whether the transparency results from specific, evolutionary adaptations as in *M. castanea*, or whether it is merely an inherent property of its very thin carapace. The thinner a material, the less light interacts with it (Fox 2010) and so many extremely small crustaceans are inherently transparent. *S. lerneri* could fall into this category.

*Skogsbergia lerneri* develops in stages (instars) of which there are five before the adult stage is reached (Cohen 1983). The purpose of the present study was to carry out a detailed examination of the ultrastructure of the adult and juvenile *S. lerneri* carapaces, and to determine how different layers of the carapace change during instar development. Two imaging techniques were used: high resolution transmission electron microscopy (TEM) and serial block face scanning electron microscopy (SBF-SEM). The latter is a new technique that allows high resolution imaging in three dimensions (Denk and Horstmann 2004).

## Materials and methods

*Skogsbergia lerneri* were collected from the east coast of the Florida Keys following the granting of Special Activity licenses (SAL-16-1796-SR and SAL-19-1796-SR) and Florida Keys National Marine Sanctuary permits (FKNMS-2016-116 and FKNMS-2018-116). They were transported to Cardiff University and maintained in purpose-built aquaria under controlled aquaculture conditions as described in detail previously (Morgan et al. 2020). Each ostracod was identified with respect to its gender and developmental stage via its shape, length, height and number of furcal claws, as categorised previously by Cohen (1983) (see Table 1).

**Table 1:** The different physical features of *S. lerneri* at each developmental stage

Instar	Length (mm)	Height (mm)	Claws on Furca
1	0.59–0.65	0.37–0.42	5
2	0.67–0.80	0.45–0.50	5
3	0.83–0.95	0.54–0.60	6–7
4	1.17–1.20 (F)	0.67–0.74	6–7
	1.01–1.20 (M)		
5	1.55–1.59 (F)	0.82–0.99	7–8
	1.26–1.47 (M)		
Adult	1.94–1.98 (F)	0.86–1.25	8
	1.50–1.73 (M)		

The length of the body becomes distinct between males (M) and females (F) when sexual dimorphism is seen at instar 4 and beyond. Table edited from Cohen (1983)

When needed, the ostracods were anaesthetised in ice-cold water for 5 min before being transferred and sacrificed in 30% ethanol. Samples were screened before selection for any observations that would indicate the ostracod was undergoing moulting or had recently done so, as mentioned in Cohen’s paper (Cohen 1983), the main indicators being a clouding of the shell, lethargy and the ostracod stopping feeding. The carapace was dissected by severing the adductor muscles and removed from the body. Single valves from each sample were fixed in modified Karnovsky fixative (2% paraformaldehyde, 2.5% glutaraldehyde) (Karnovsky 1965) and washed in 0.1 M sodium cacodylate. Ostracods were then prepared for SBF-SEM and TEM with the Deerinck method (Deerinck et al. 2010) using 1% osmium tetroxide/1.5% potassium ferricyanide added to samples for an hour. Samples were then immersed in 60 °C 1% thiocarbohydrazide for 20 min, 1% osmium tetroxide for one hour, 1% aqueous uranyl acetate overnight before being stained with Walton’s lead aspartate (Walton 1979) for 1 hour at 60 °C. Valves were washed in distilled water three times for 10 min each after every step. Then tissue was serially dehydrated through 70–100% ethanol for 15 min each and then further dehydrated in 1:1 ethanol:acetone and then 100% acetone twice for 15 min each.

Samples were infiltrated and embedded in Durcupan resin (Cat: 44610 Sigma Aldrich, MO, USA) according to manufacturer’s instructions; carried out in steps of acetone:Durcupan (1:1) and (1:3) for 2 hours each before being placed in 100% Durcupan overnight. Durcupan was then renewed twice over 6 h and samples were individually embedded into moulds. Embedded samples were then polymerised at 60 °C for a minimum of 24 h. Once fully hardened, samples were trimmed into a small trapezoid shape and polished via cutting of the sample block’s surface with a glass knife until it became a smooth surface. For image analysis, samples were always generated from the same area of the carapace, the carapace was cut to a third of the length from the anterior of the carapace, to generate similar readings across all results.

For SBF-SEM, samples blocks were trimmed further, and the surface was cut into a 1 × 1 mm square before being gold-coated at 7 nm thickness. Serial 50 nm cuts were carried out by the 3view ultramicrotome and the block face was imaged using the Gatan 3view 2XP system at 4 × 4 K with a pixel resolution of 7.5 nm per pixel and a pixel dwell time of 8 µs. Approximately 1000 serial images per tissue block were taken and imported into Amira for life Sciences 6.0.1 (ThermoFisher Scientific, MA, USA). For TEM, 100 nm ultrathin sections were cut from the SBF-SEM sample blocks, placed on grids and analysed on a Jeol 1010 microscope at 80 kV.

Ultrastructural analysis of every development stage (multiple measurements from *n* = 5 ostracod samples) was carried out with TEM, and data-intensive 3D models were generated by SBF-SEM (*n* = 1) at each stage. All comparisons of size differences between the instars were carried out via one-way ANOVA testing with Tukey's post hoc, calculated with SPSS, showing significance at *P* < 0.05. Direct comparisons between two sets of data were carried out with independent *t*-testing.

## Results

**Fig. 2:**
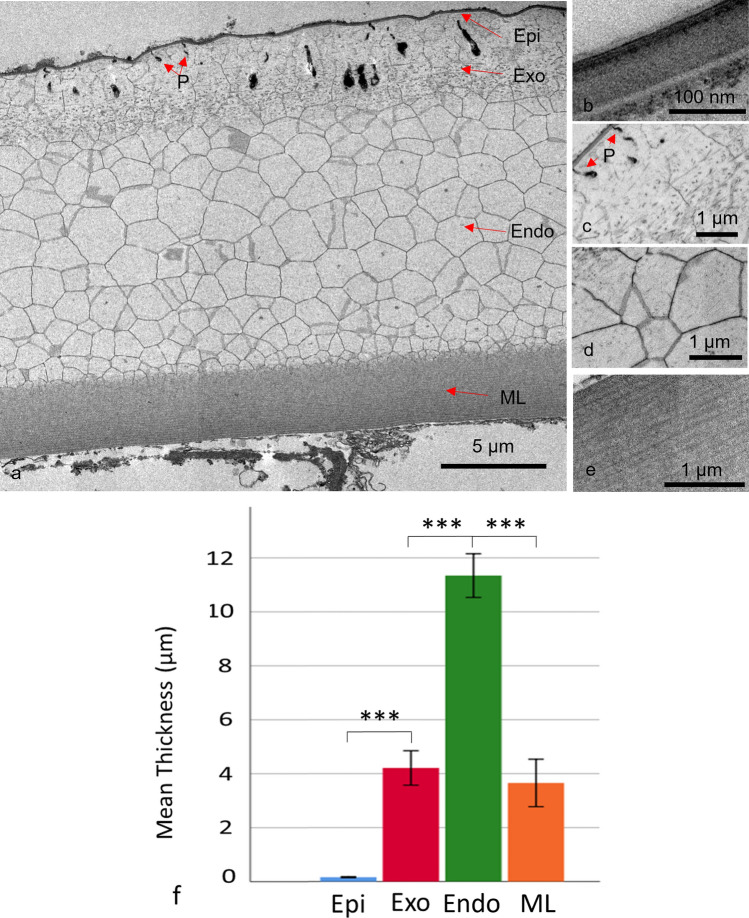
**a** A transmission electron microscopy cross-section of the adult *S. lerneri* carapace. The carapace is separated into four major layers: the epicuticle (Epi), the exocuticle (Exo), the endocuticle (Endo) and the membranous layer (ML). Examples of epicuticle projections are labelled with P. The individual images are shown for each of the four major layers **b** the epicuticle, **c** the exocuticle, **d** the endocuticle, **e** the membranous layer. **f** Measurements of the adult carapace showing the thickness of each layer (number of animals used, *n* = 5). *** Represents *P* < 0.001. Error bars are ± SD

TEM images of the adult carapace showed a layered structure, namely epicuticle, exocuticle, endocuticle and membranous layer (Fig. 2a–e), consistent with other myodocopid ostracods, with a mean thickness of 19.2 ± 1.78 µm, *n* = 5.

Significant differences in thickness (ANOVA, *F* (1,25), all *P* values were *P* < 0.001) were seen between all the layers except between the exocuticle and membranous layer (Fig. 2f) reflecting their different contributions to the overall mechanical and optical properties of the adult carapace. Chitin fibrils were present in the exocuticle and the membranous layer, although they appeared less dense and disorganised in the exocuticle (Fig. 2c) compared to the regular lamellae of chitin fibrils in the membranous layer (Fig. 2e).

**Fig. 3:**
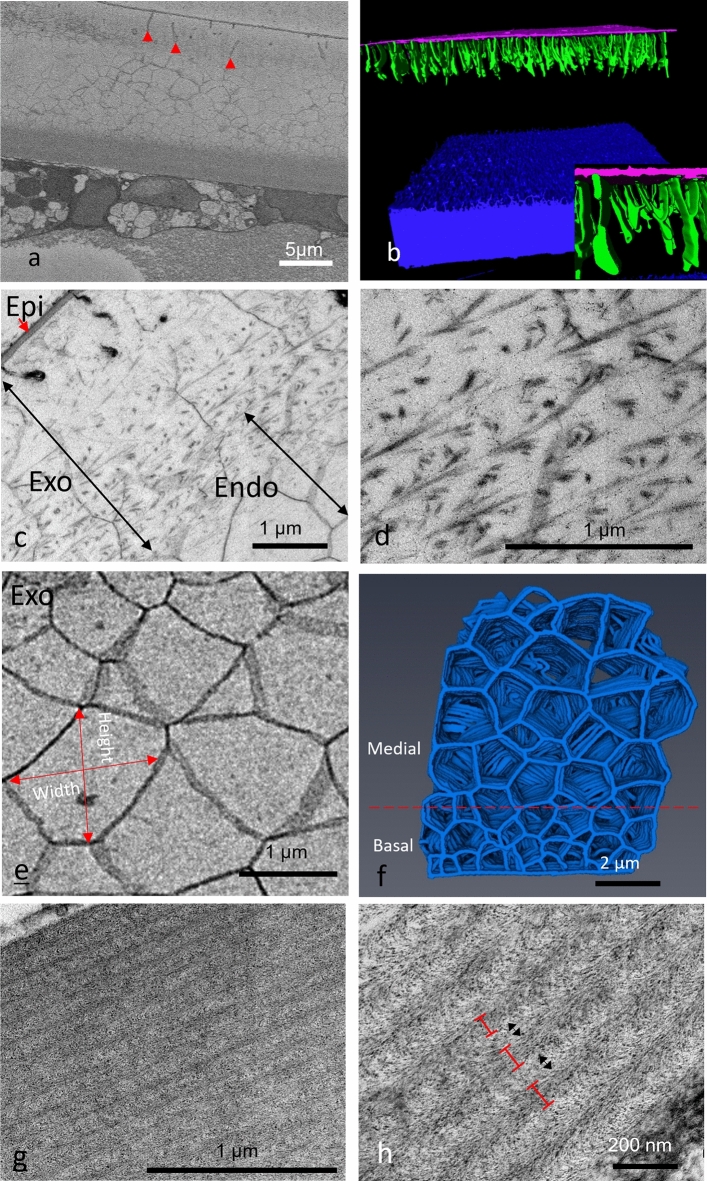
**a** A representative serial block face scanning electron microscopy (SBF-SEM) image of the adult *S. lerneri* carapace with red arrowheads indicating epicuticle projections and **b** manually segmented epicuticle (pink) projections (green) within a 3D reconstruction of SBF-SEM serial images. The membranous layer is shown in blue, with the exocuticle and endocuticle not shown. The full 3D reconstruction can be seen in online resource 3. An enlarged view of the projections is in the inset, where these projections can be seen to become larger at the foot. **c** Transmission electron microscopy (TEM) micrographs of the adult exocuticle, between the epicuticle and endocuticle, seen at ×3000 and **d** ×10,000 magnification. The exocuticle and endocuticle merge with each other, as indicated by the overlapping, double-headed arrows in **c**. Some of the fibrils appear to be roughly parallel to the epicuticle surface and display curved ends, as seen in **d**, but this may depend on how the fibrils are oriented with respect to the section plane. **e** A representative SBF-SEM image of the endocuticle showing the crystal dimensions. **f** A 3D manual reconstruction of the SBF-SEM data, showing the medial and basal crystals within the adult. **g** TEM image of the membranous layer at ×4000 and **h** ×10,000 magnification. Chitin lamellae are identified via the red bars and the inter-lamellae spaces are shown via the black double-headed arrows. *Epi* epicuticle, *Exo* exocuticle and *Endo* endocuticle

The epicuticle was seen to consist of three distinct parts, a thin apical layer, a thicker medial layer and an electron dense basal layer (Fig. 2b and Online Resource 1). The thickness of the epicuticle did not vary across the carapace section and the separation between the epicuticle and exocuticle was clearly distinct. Pore canals (Online Resource 1) and epicuticle projections (Fig. 2a, c) were seen travelling from the epicuticle through the exocuticle in TEM imaging. This was also seen in SBF-SEM, where the pore canals were particularly visible (Online Resource 2). These pore canals can be seen in the 3D reconstructions to twist throughout the carapace. The projections were much smaller and more numerous than the pore canals and stopped before they reached the calcified layer. These projections commonly branch closer to the epicuticle and become larger at the foot of the projection. While these projections appeared sparse in 2D TEM micrographs (Fig. 2) and individual SBF-SEM slices (Fig. 3a), 3D SBF-SEM reconstructions show that they densely populate the carapace (Fig. 3b) (Online Resource 3).

The adult exocuticle showed a series of chitin fibrils much more loosely packed compared to the membranous layer but nevertheless showing a degree of organisation (Fig. 3c, d). The distinctly curved shape of the individual fibrils shows the presence of a twisted plywood structure within the layer. Unlike the epicuticle/exocuticle interface, the exocuticle/endocuticle interface is hard to define and the layers appear to merge with each other (Fig. 3c).

The endocuticle contained polyhedrons of differing sizes (Fig. 3e), which represent the expected calcium carbonate crystal structures observed in other myodocopid ostracods (Yamada 2019), outlined by an organic matrix. The polyhedrons were split into larger crystals near the centre and smaller, more numerous ones near the base (Fig. 3f). Medial crystalline polyhedrons (mean height: 1.07 ± 0.3 µm, *n* = 5 ostracods width: 1.02 ± 0.34 µm, *n* = 5) were significantly larger, than the basal polyhedrons (mean height: 0.34 ± 0.1 µm, *n* = 5 width: 0.39 ± 0.13 µm, *n* = 5), both in height (ANOVA, *F* (1,46) = 82.66, *P* = 0.002) and width (ANOVA, *F* (1,46) = 97.92, *P* < 0.001). With height being defined as the vertical distance taken from the basal end of the crystal to the apical from the cross section and width the horizontal distance.

The membranous layer was composed of the more traditional organised rows of chitin lamellae (Fig. 3g, h). As with the chitin fibrils in the exocuticle, the ends of the individual fibrils can be seen to curve, suggesting a similar twisted plywood formation in the membranous layer. The mean thickness of each lamella was measured by eye in triplicate for every sample and found to be 75.9 ± 13.7 nm, *n* = 5 ostracods with a significantly smaller (Paired sample *t* test (26) = 15.84, *P* < 0.001) distance of 44.05 ± 4.4 nm, *n* = 5 between them, measured from the edge of one lamella to the near edge of its neighbour. The mean number of lamellae contained within the adult membranous layer was 17.6 ± 4.6, *n* = 5 and ranged between 12 and 24.

## Instar development

**Fig. 4:**
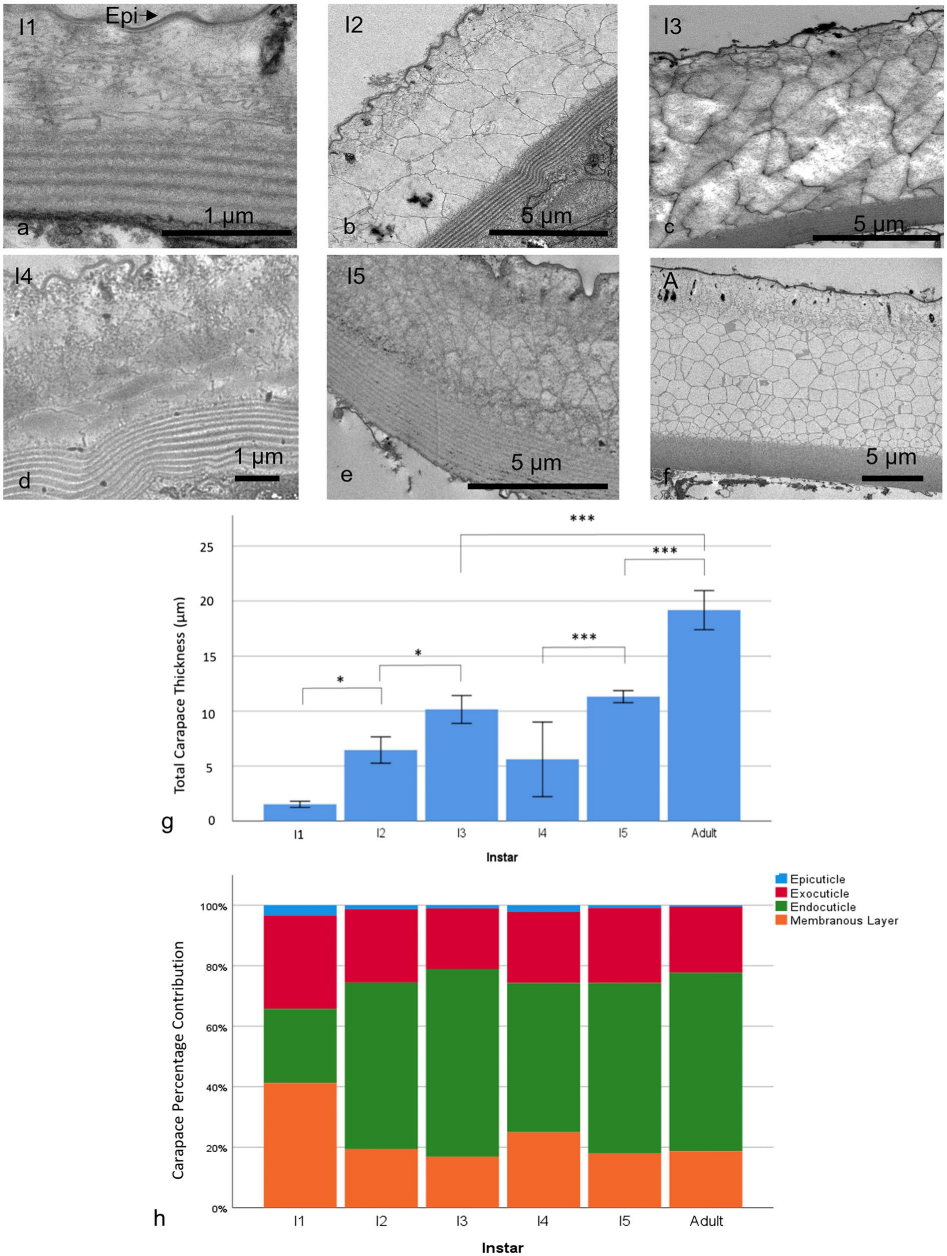
Transmission electron microscopy cross sections of the carapace from each instar, **a** instar 1 (I1), **b** instar 2 (I2), **c** instar 3 (I3), **d** instar 4 (I4), **e** instar 5 (I5) and **f** the adult (A) for reference. **g** The mean carapace thickness at each instar, number of animals used, *n* = 5. **h** The percentage proportions of the layers within each instar. * Represents *P* < 0.05 and *** is *P* < 0.001. Error bars are ± SD

As the instars developed, the carapace became thicker and each of the layers became more defined, especially in the early instars (Fig. 4a–f). Only a rudimentary endocuticle could be seen before instar 2.

To quantify these changes, the total carapace thickness was measured for each instar (Fig. 4g). Instar 2 showed significant growth of 5 µm in carapace thickness (ANOVA, *F* (5,22) = 55.36, *P* = 0.04). Instar 3’s carapace was also significantly thicker than its predecessor (*P* = 0.03) with a mean thickness of 10.16 ± 1.27 µm, *n* = 5. There appears to be a drop in thickness from instar 3 to instar 4, however, due to a high standard deviation in the statistics no significant change could be stated. The carapace of instar 5 was significantly thicker than that of instar 4 (*P* = 0.001). Finally, the adult carapace was significantly thicker than all other instars (*P* < 0.001) (Online Resource 4). Figure 4h shows the contribution of each layer as a percentage of carapace thickness. The ultrastructural proportions changed most drastically at early instars and this was caused by a disproportionate increase in the endocuticle (Online Resource 5).

The epicuticle thickened from the first instar to the third at 100.66 ± 9.6 nm, *n* = 5 (ANOVA, *F* (5,24) = 6.378, *P* < 0.001), then maintained a similar thickness with all further instars being significantly larger than instar 1 but not significantly different to each other.

**Fig. 5:**
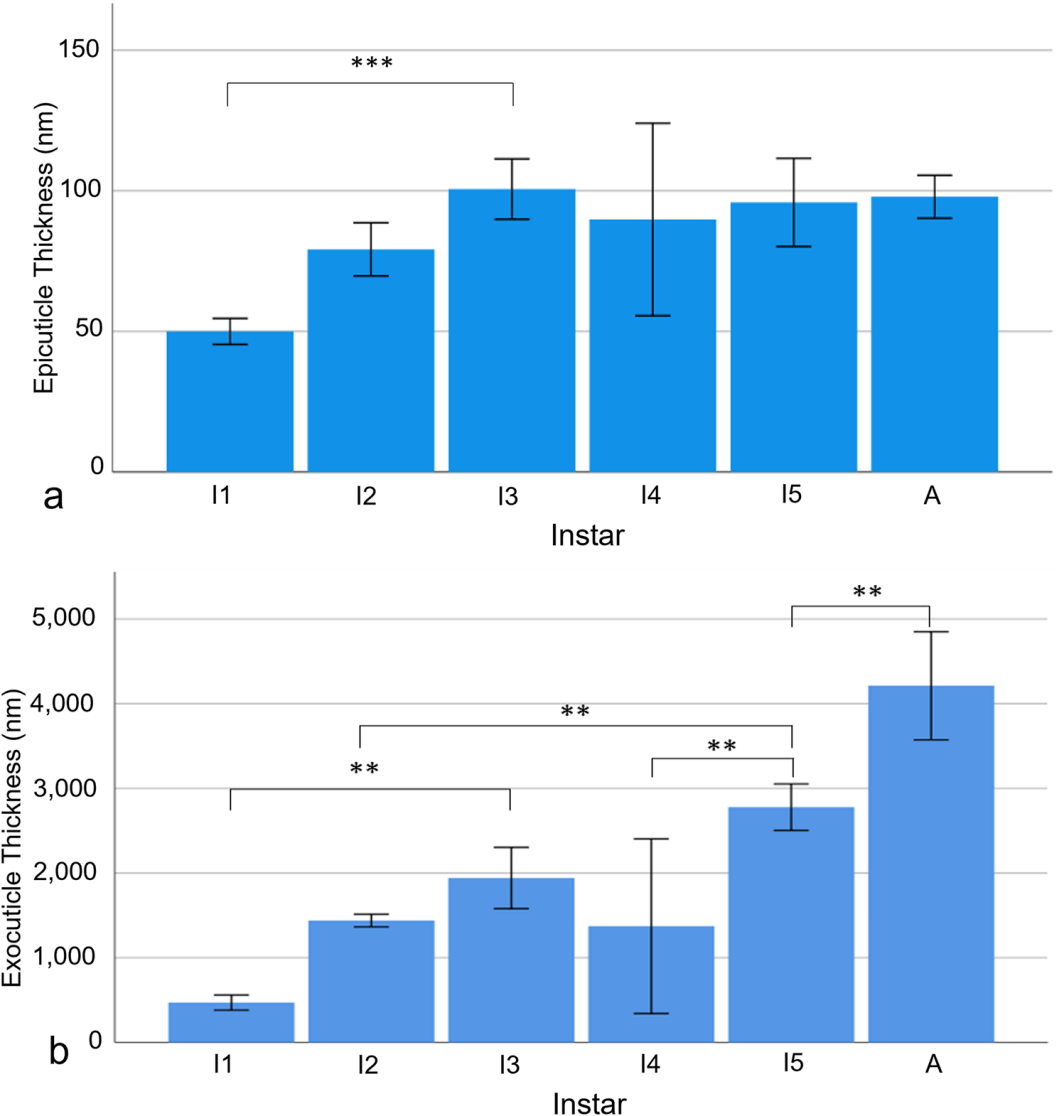
The thicknesses of the **a** epicuticle (number of animals used, *n* = 5) and **b** the exocuticle (*n* = 5) throughout development. ** Represents *P* < 0.01 and *** represents *P* < 0.001. Error bars are ± SD

The exocuticle thickness grew as a function of increasing developmental stages, with the exception of instar 4 (Fig. 5b) (Online Resources 5 and 6). The increase in exocuticle thickness was significant between instar 1 and 3 (ANOVA, *F* (5,22) = 27.64, *P* = 0.006), instar 4 and 5 (*P* = 0.009) and instar 5 and the adult (*P* = 0.005), which was thicker than all other instars. However, as in the epicuticle, instar 4 thickness, though not significant, appeared to decrease after instar 3.

**Fig. 6:**
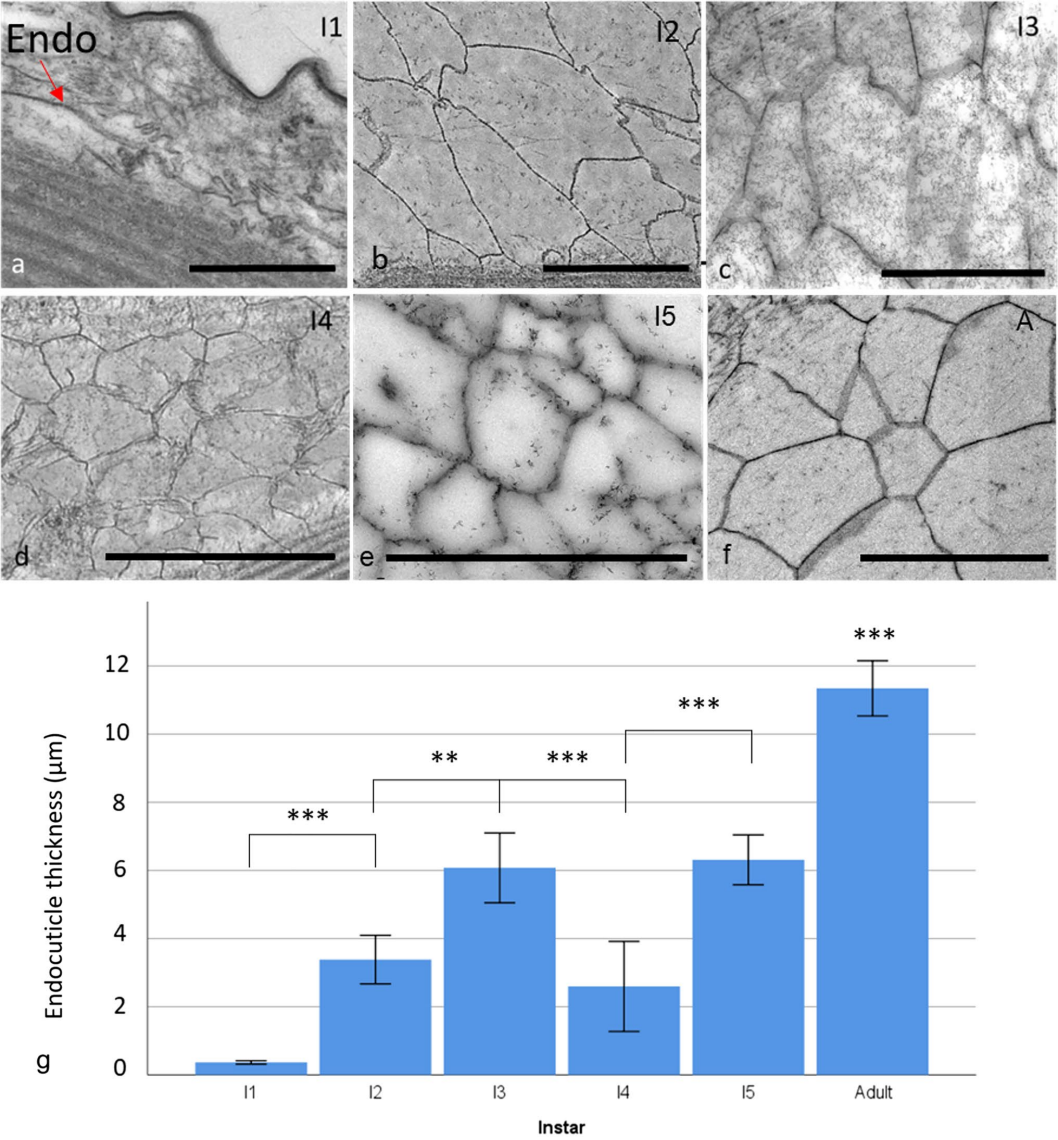
Transmission electron microscopy images of the endocuticle through each developmental stage showing **a** instar 1 (the undeveloped layer identified by Endo), which lacked fully formed crystals, **b** instar 2, **c** instar 3, **d** instar 4, **e** instar 5 and **f** the adult. Scale bar is 2 μm. **g** The endocuticle thickness across instar development (number of animals used, *n* = 5). ** Represents *P* < 0.01 and *** represents *P* < 0.001. Error bars are ± SD

The endocuticle increased sharply at the beginning and the end of *S. lerneri* development, with more gradual progression during the intermediate stages (Fig. 6). The barely visible endocuticle of instar 1 became significantly thicker (ANOVA, *F* (5,22) = 77.762, *P* = 0.001) in instar 2. The endocuticle then increased again (*P* < 0.001) at instar 3. The instar 4 endocuticle decreased in thickness, however, unlike the previous carapace layers, this decrease was significant (*P* < 0.001). This layer increased at instar 5 (*P* < 0.001) and the adult endocuticle was significantly thicker than every other instar.

Every instar with fully developed crystals had significantly larger medial polyhedrons and smaller basal ones, both in height and width (Online Resource 7). Instar 4 surprisingly followed the same pattern as the other instars within the calcified layer, containing similarly sized basal polyhedrons as well as larger medial polyhedrons (Paired *t* test, *t*8 = 4.88, *P* = 0.002). This was counter to the pattern of instar 4 development in previous carapace layers, where instar 4 had shown a decrease in size. While there were significant differences between the differently positioned polyhedrons in each instar, the only significant difference between polyhedrons of the same position throughout the instars, was between instar 5 and the adult. Although the endocuticle was large in the adult, the polyhedrons were in fact smaller. The medial polyhedrons had a significantly smaller height (ANOVA, *F* (4,23) = 3.82, *P* = 0.016) and the basal ones had significantly shorter widths (ANOVA, *F* (4,23) = 4.58, *P* = 0.003). When the medial and basal crystals were averaged together, the crystals were roughly equal in diameter; except for in instar 1, no significant difference was seen between height and width in the polyhedrons within each instar (Online Resource 8). This is especially true for the adult crystals where the difference was only 0.05 µm, this translates to a 0.03% difference between them.

**Fig. 7:**
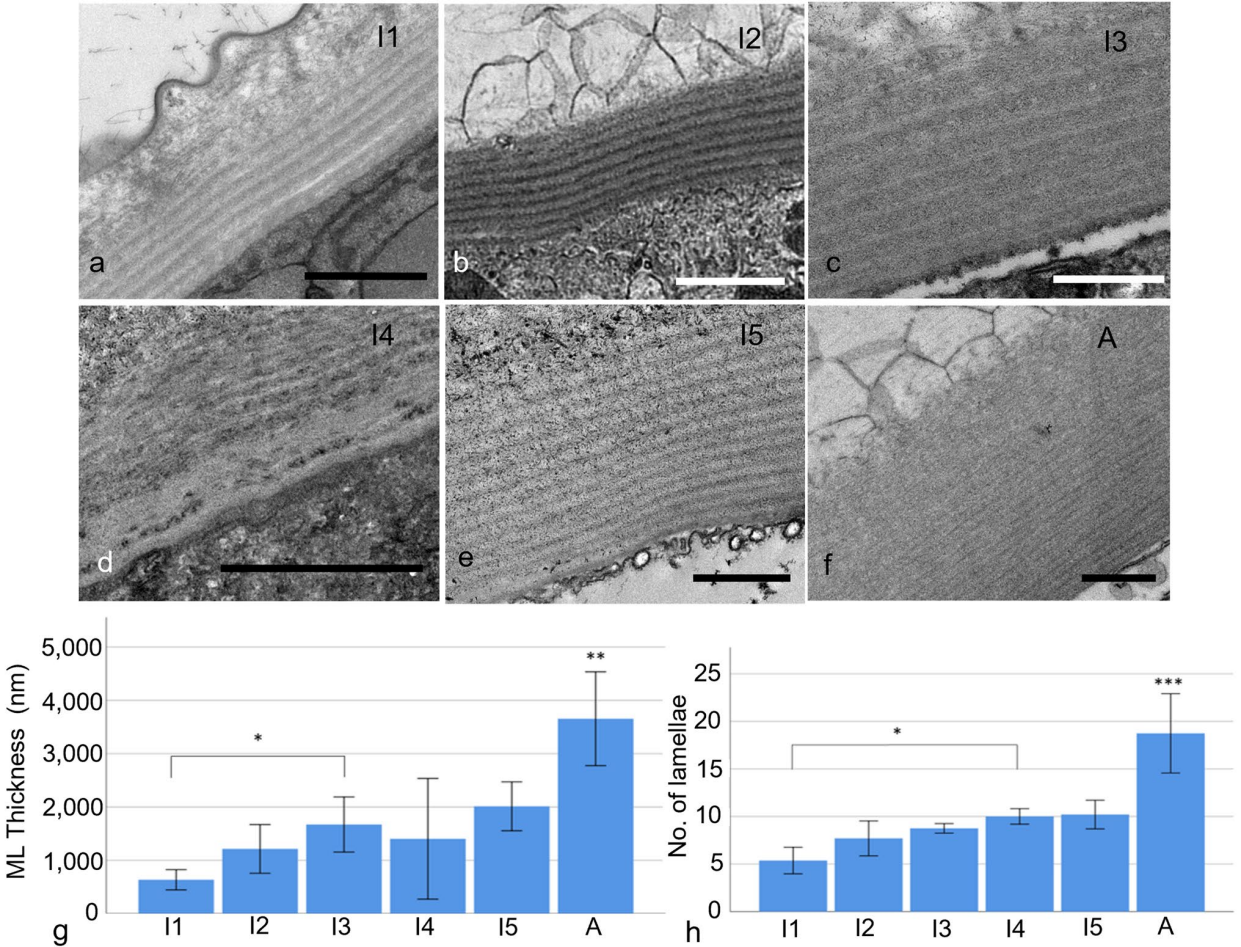
Transmission electron microscopy images of the membranous layer from **a** instar 1, **b** instar 2, **c** instar 3, **d** instar 4, **e** instar 5 and **f** the adult. The individual chitin lamellae can be seen within all instars. **g** The thickness of the membranous layer (ML) throughout development (number of animals used, *n* = 5). **h** The number of lamellae within the membranous layer throughout development (*n* = 5). Scale bars are 1 μm.* Represents *P* < 0.05, ** represents *P* < 0.01 and *** represents *P* < 0.001. Error bars are ± SD

The membranous layer appeared to grow gradually thicker throughout development, and sharply in the adult (Fig. 7a–f). A significant increase in membranous layer thickness was seen at instar 3, the thickness being greater than instar 1 (ANOVA, *F* (5,18) = 39.23, *P* = 0.019). The adult membranous layer was significantly thicker than the rest (*P* < 0.01) (Fig. 7g).

The number of lamellae also increased as the ostracod developed (Fig. 7a–f, h); this increase was gradual until a sharp increase in the adult, which was even more distinct than that seen in the membrane layer thickness. The first significant increase was seen between instar 4 and instar 1 (ANOVA, *F* (5,21) = 23.02, *P* < 0.049) which was counter to the trend seen in the other layers of instar 4 and the second was a more significant increase between instar 5 and the adult (*P* < 0.001).

The thickness of the individual chitin lamellae within the membranous layer was always significantly greater than the inter-lamellar separation. There were very few differences between the lamellae at each instar, the thickness of the instar 3 lamellae was significantly less than that of instar 5 (ANOVA, *F* (5,21) = 3.87, *P* = 0.013) and instar 2 (*P* = 0.03), and the distance between the instar 3 lamellae was significantly less than that in instar 5 (*P* = 0.009) (Online Resource 9).

## 3D reconstruction of carapaces throughout the instars

3D reconstructions of carapaces were examined to further interrogate carapace structure, and to confirm the changes seen in 2D images. Additionally, the structure of the developing calcified endocuticle was visualised; this layer is of particular importance because its calcification significantly affects the overall mechanics of the carapace.

**Fig. 8:**
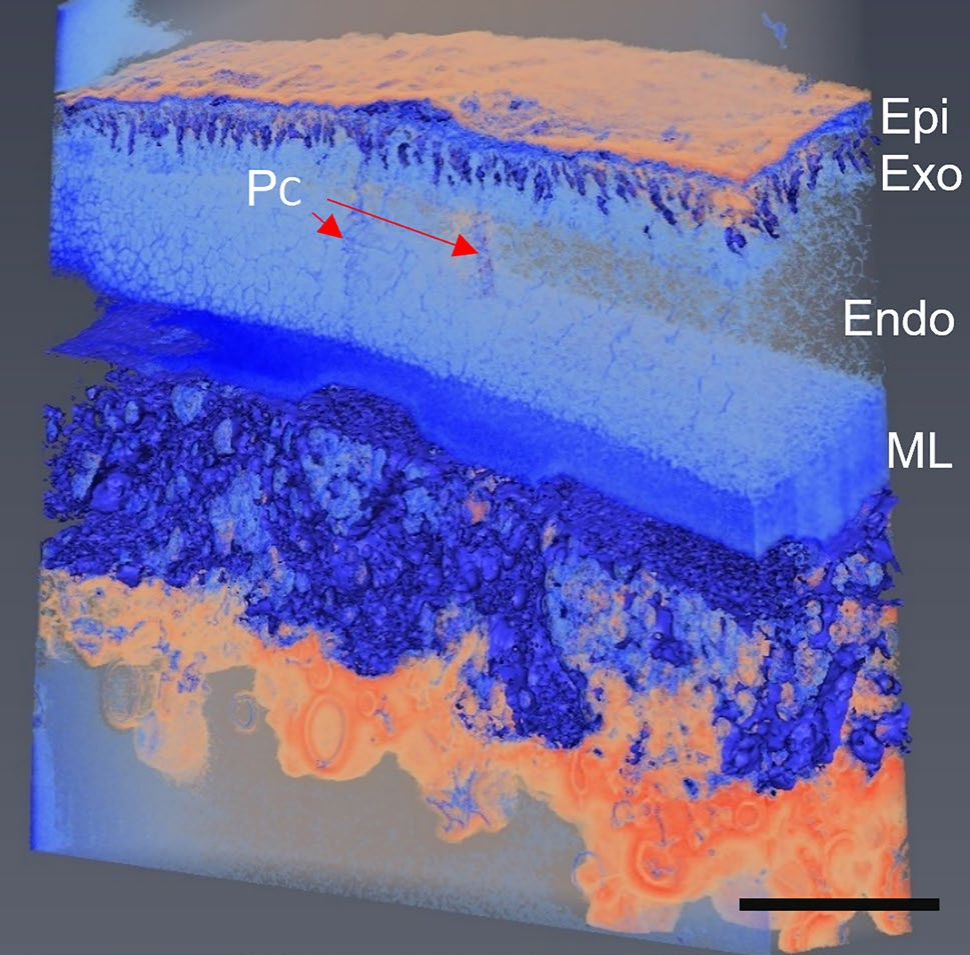
3D volume render of an adult carapace generated via serial block face scanning electron microscopy. The calcified layer was made more transparent so the pore canals can be seen. Apparent gaps seen in the endocuticle are a result of incomplete staining leading to a lack of contrast. Colours are due to the automated thresholding used. *Epi* epicuticle; *Exo* exocuticle; *Endo* endocuticle; *ML* membranous layer; *PC* pore canal. Scale bar is 10 μm

The 3D model, consistent with the 2D imaging data, showed the constant proportions of the layers along the length of the carapace in the adult ostracod (Fig. 8); the same result was seen in all developmental stages (Online Resource 10). The twisted nature of the pore canals as they traverse through the carapace becomes clear in these reconstructions (Fig. 8 and Online Resource 2). The layers remained parallel and intact throughout the thickness implying the 2D cross sections provided a good representation of the carapace. 3D reconstructions were made of carapace sub-volumes for each instar to observe changes across development. Analysis of the 3D reconstructions showed no inconsistencies with the 2D TEM imaging as the proportions of the layers remained constant and mostly parallel to each other.

**Fig. 9:**
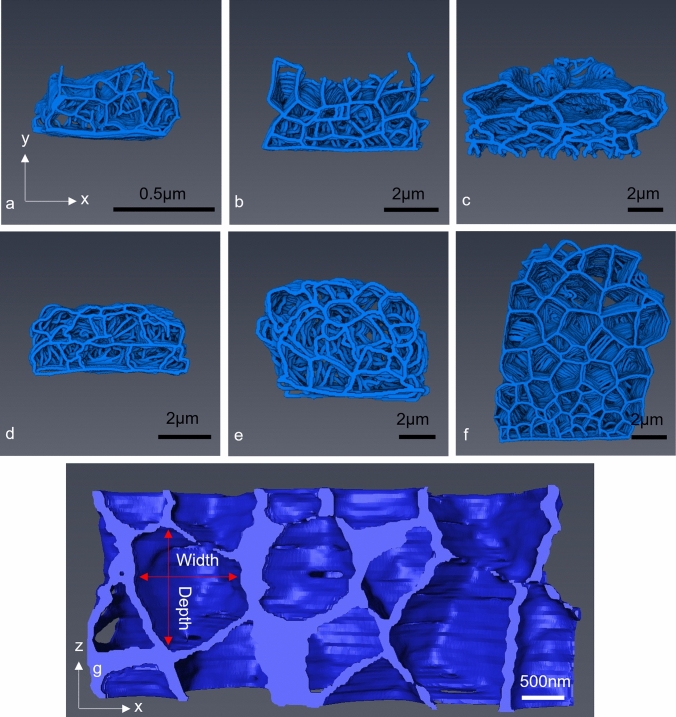
Manually segmented 3D models of transverse sections of the endocuticle at each developmental stage, generated from SBF-SEM data: **a** instar 1, **b** instar 2, **c** instar 3, **d** instar 4, **e** instar 5 and **f** the adult. **g** A horizontal cross section through the adult endocuticle interpolated across the individual *x*–*y* images (hence the lower resolution than in the transverse reconstructions). The shape of the crystalline polyhedrons can be seen to be as disorganised and variable as in the transverse plane. Arrows represent previously measured width and new *z*-axis depth for orientation

The endocuticle layer was further analysed in isolation of other components of the carapace (Fig. 9), to study in greater detail the organic matrix that outlines the crystal structures. The 3D analysis revealed that the polyhedrons were unorganised and of variable size in the *z*-axis through the carapace, as well as along the face of the transverse sections (Fig. 9).

## Discussion

Changes in myodocopid ostracod development across the instars have been analysed previously; these were measurements recorded from a surface view, either using SEM or observations by eye (Kesling 1951; Cohen 1983; Yumoto 1994) or are focused on the changes in the overall size of the ostracod body (Gerrish and Morin 2008; Pereira et al. 2017). Using scanning electron microscopy (SEM), Sohn and Kornicker (1988) studied the calcified cuticle of myodocopids, including *Vargula hilgendorfii* (Mueller, 1890), and developmental change in uncategorised juveniles. The microstructures of some of these calcified cuticles were similar to *S. lerneri*. However, the present study was the first to quantify developmental changes across every stage of development in the ultrastructure of the myodocopid carapace at high resolution using the volume electron microscopical technique of SBF-SEM.

The *S. lerneri* carapace thickened throughout the instars until the adult stage was reached but this increase in thickness was not proportional to the overall change in size of the carapace. Throughout its lifetime, the length of the *S. lerneri* carapace increases by a factor of 3.4, from 0.6 to 2.0 mm (Cohen 1983), whereas the mean thickness increase found in the present study was from 1.5 to 19.2 µm, a factor of 12.7, similar to that seen in the podocopid *Xestoleberis hanaii* Ishizaki (Yumoto 1994). This difference could arise from a need to strengthen the carapace in the adult by reducing the in-plane stress as the carapace develops. If approximated to a thin spherical shell, the in-plane stress is proportional to the ratio diameter/thickness, which means that the adult will have approximately a quarter (3.4/12.7) of the stress compared with instar 1.

The epicuticle is known to help maintain correct hydration within the organism (Price and Holdich 1980); other functions are not fully understood but, being the outermost layer, the epicuticle likely serves a protective role. *S. lerneri is* a benthic ostracod (Kornicker et al. 2002) and spends much of its time in the sand, so this protective function would include prevention of abrasion and its associated detriment to optical transparency. There was a doubling in epicuticle thickness over the ostracod development and the structure did not change throughout. The epicuticle ultrastructure was similar to other myodocopids such as the philomedid *Euphilomedes japonica* (Mueller 1890) (roughly similar in size to *S. lerneri*), and the cypridinids *V. hilgendorfii* (around 2–3 mm long) (Ogoh and Ohmiya 2005) and *Melavargula japonica* (Poulsen, 1962) (Yamada 2019). The chitin-containing exocuticle and membranous layer progressed similarly throughout development. They both increased in thickness as the instars progress, the exocuticle became nine times thicker from instar 1 to adult, and the membranous layer six times thicker. These large increases are actually lower than the total thickness increase, so their combined contribution to the overall thickness decreases during development. This is primarily because the endocuticle increases disproportionally (occupying 24% of the carapace in instar 1 compared to 59% in the adult). From instar 2 onwards, the total proportion of the carapace stayed between 22–24.5% and 16.5–25% for the exocuticle and membranous layer, respectively.

The exocuticle contains chitin fibrils organised much more loosely than those in the membranous layer, which is composed of regularly spaced chitin lamellae. Both layers showed a chitin orientation parallel to the surface, similar to other ostracod carapaces such as* Cypridopsis vidua* (Bate and East 1972). The chitin showed patterning representative of the typical twisted plywood structure (Bouligand 1972). *S. lerneri*’s chitin organisation closely resembled that seen within most ostracods (Bate and East 1972; Smith and Bate 1983). The highest degree of similarity is seen within the same order, myodocopida (Yamada 2019), with the total thickness in the adult *S. lerneri* being consistent with the other exocuticle thicknesses seen, although slightly thinner than that of the non-transparent *E. japonica* (Yamada 2019).

The individual *S. lerneri* lamellae within the membranous layer do not grow in size as the ostracod develops. The increase in membranous layer thickness therefore arises from an increased number of lamellae as the ostracod grows, suggesting that there is an optimal thickness for the lamellae to function. The exception to this was instar 3, whose lamellae were significantly thinner than those in instars 2 and 5, with an inter-lamella distance smaller than instar 5. The lack of other changes in size suggests that this is not a developmental effect but is instead instar 3 specific. However, it is possible that some of the instar 3 carapaces were caught near post-moult, which would not be identifiable by the naked eye, and were thus not representative of that particular instar. Chitin lamellae in other myodocopids only develop post-moult (Yamada 2019) and seem to be thinner while developing. Significant differences seen in every instar between the inter-lamellae distance and lamella thickness implies that the layer formation is highly regulated. This inter-lamellae distance changes alongside the lamella thickness so that the spacing is always around 56.5–66.5% of the lamella thickness, again showing a high level of regulation.

TEM sections showed there to be about 20 chitin lamellae, organised and spaced in a similar pattern in the adult. Lamellae number has previously been linked to calcification level and carapace weight (Voss-Foucart and Jeuniaux 1978), so large lamellae numbers may be indicative of strong calcification. Similarity seen between *S. lerneri* and *V. hilgendorfii* (Yamada 2019) may be due to their comparatively close taxonomy. In contrast, the podocopid ostracod *Bicornucythere bisanensis* (Okubo, 1975) possesses a very thin chitinous endocuticle (Okada 1982) although it still possesses significant calcification. Laminar structures are well known to increase material strength, especially bending resistance (Fabritius et al. 2009).

As seen in our results, the proportion occupied by the endocuticle increases with development. Calcification increases the hardness of tissues, so the increased mineral uptake throughout the ostracod’s lifespan would confer significant additional rigidity as the carapace develops. The calcified endocuticles of ostracods are likely to consist of calcium carbonate polymorphs (Kesling 1951; Jorgensen 1970; Rosenfeld 1979). Using SEM, Sohn and Kornicker (1988) showed a range of ultrastructures in various myodocopids, of which several features have also been seen in our results, including “coarse granular” minerals in the endocuticle. The *S. lerneri* ultrastructure was also similar to that of *V. hilgendorfii* (Yamada 2019), seen via TEM, with the polyhedrons shrinking in size proximal to the lamellae. We found that this change was seen throughout the entire development, a reduction of approximately a third in all instars. No significant change in the size of the polyhedrons was seen throughout the ostracod’s lifespan, until the adult stage, where they decreased in size compared to instar 5. This meant thicker layers were made up of more polyhedrons instead of larger ones, which may suggest an imposed polyhedron size limit. Instar 1 contained an undeveloped endocuticle, which is most likely due to the ostracod having limited access to the various minerals or available energy within the egg. The *S. lerneri* polyhedrons had no significant difference between the height and the width and no preferred morphological orientation, which is similar to other myodocopida. Podocopid ostracods have much larger crystals with no morphological orientation, which decrease in size towards the base (Jorgensen 1970). These crystal units range from 6 to 0.5 µm at the basal layer. Therefore, if there is an imposed maximum size limit on the polyhedrons within *S. lerneri*, it may be restricted to the myodocopid order. The podocopid basal layer crystal size of 0.5 µm however, is close to that within *S. lerneri* (0.52 µm) so there may be a minimum crystal size limit across ostracods. However it is worth noting that ostracods may be polyphyletic and so podocopids may not be closely related to myodocopids (Siveter et al. 2010).

Analysis of some podocopid ostracods has shown these carapaces possess amorphous calcite which gradually transforms into calcite crystallites, except in weakly calcified ostracods (Keyser and Walter 2004). However the polyhedrons of the myodocopid *V. hilgendorfii* seem to not be comprised of the expected calcite but consist of amorphous calcium carbonate or monohydrocalcite (Yamada 2019). Yamada (2019) suggests that *V. hilgendorfii* is nekto-benthic and so spends more time away from the ocean floor than benthic species; this can explain the lightweight carapace, with pelagic species reducing their weight even further by possessing very little mineralisation, if any. S. *lerneri* being benthic (Kornicker et al. 2002) reinforces this idea, maintaining a thicker carapace than *V. hilgendorfii* and an equal, if not higher, level of mineralisation, comprising amorphous calcium carbonate and aragonite (unpubl data).

Although the primary role of the ostracod carapace is mechanical, the *S. lerneri* carapace appears transparent to the human eye. In the absence of pigment, several different mechanisms can eliminate light scattering and produce transparency, and it is hard to identify which is used by *S. lerneri*. A material’s thickness has a substantial effect on its ability to become transparent; the linear attenuation coefficient from the Beer–Lambert law for solids (Swinehart 1962) shows that the thinner the material, the lower the impact of scattering/absorption. As such, the thinner a material becomes, the easier it is for that material to maintain transparency. Thus, the thin carapace in *S. lerneri* (with a mean thickness in adult carapaces of 19.2 µm) may contribute towards its transparency. Another method for a material to be transparent is when all its components have the same refractive index (RI) as is seen in glass (Maurice 1957). While it is highly unlikely that every layer of the *S. lerneri* carapace has the same RI, examples have been seen of structures organised to moderate a sharp RI change into a smoother one, creating less scattering (Born and Wolf 1980). The projections coming down from the epicuticle, the absence of a distinct exocuticle–endocuticle border and the decrease of polyhedron size close to the membranous layer, all suggest the presence of gradients between adjacent layers to minimise changes in RI. These specific ultrastructural features may also contribute towards the transparency of the *S. lerneri* carapace.

One of the most exciting aspects of this data is the application of serial block face SEM for the creation of complete 3D reconstructions. This creates a model that possesses a resolution and contrast comparable to TEM imaging, while also not being limited to a surface view like standard SEM. Similar data results could only previously be generated via the extremely difficult and time-consuming process of serial TEM (Birch-Andersen 1955; Bang and Bang 1957). Through the use of this new method, we were able to capture the pore canal in 3D space and manually map the twisting path it takes throughout the carapace (online resource 2). It also allowed us to identify the honeycomb nature of the calcified structure through tracing of the organic matrix that surround the mineral formations. The combination of this SBF-SEM and regular TEM imaging has thus helped to create a comprehensive view of the *S. lerneri* carapace.

## Supplementary Information

Below is the link to the electronic supplementary material.

Supplementary file1 (PDF 232 KB)

Supplementary file2 (AVI 88424 KB) **Online Resource 2** A video showing a serial stack fly through of 1000 slices of the adult carapace with the pore canal highlighted in red, followed by a rotation of the carapace generated with an automated volume render, showing the pore canal within. The pore canal can be seen to twist and stretch throughout the carapace.

Supplementary file3 (MPG 437493 KB) **Online Resource 3** A video showing a manually segmented 3D reconstruction identifying the epicuticle (pink), lamellar endocuticle (blue) and the epicuticle projections (green).

Supplementary file4 (PDF 131 KB)

Supplementary file5 (PDF 129 KB)

Supplementary file6 (PDF 260 KB)

Supplementary file7 (PDF 128 KB)

Supplementary file8 (PDF 201 KB)

Supplementary file9 (PDF 127 KB)

Supplementary file10 (MP4 857077 KB) **Online Resource 10** A video showing a serial stack fly through of 1000 slices of the carapace, followed by a rotation of an automated volume render for **a** instar 1, **b** instar 2, **c** instar 3, **d** instar 4, **e** instar 5 and **f** the adult.

## Data Availability

The datasets generated during and/or analysed during the current study are available from the corresponding author on reasonable request.
