# Supplementary material for: The ultrastructural development and 3D reconstruction of the transparent carapace of the ostracod Skogsbergia lerneri
Source: Mar Biol. 2022 Feb 13;169(3):35. doi: 10.1007/s00227-021-04006-7 (PMC8841342; doi:10.1007/s00227-021-04006-7)
Supplement: Supplementary file 4 — Supplementary file4 (PDF 131 KB) [file 227_2021_4006_MOESM4_ESM.pdf]

Online Resource 4 The changes in ultrastructure across development in respect to their overall thickness and the approximate proportions of each layer (number of animals used, n = 5 for each instar)

Instar	Thickness (µm)	Epicuticle (%)	Exocuticle (%)	Endocuticle (%)	Membranous Layer (%)
1	1.5 ± 0.24	3.37 ± 0.62	30.90 ± 1.62	24.46 ± 3.60	41.27 ± 6.30
2	6.5 ± 1.20	1.34 ± 0.26	24.17 ± 3.41	55.11 ± 3.15	19.37 ± 4.19
3	10.16 ± 1.27	1.05 ± 0.17	20.06 ± 4.12	62.00 ± 7.49	16.89 ± 3.49
4	5.61 ± 4.01	2.14 ± 0.94	23.56 ± 4.68	49.3 ± 3.39	25.00 ± 6.65
5	11.3 ± 0.55	0.86 ± 0.16	24.82 ± 2.29	56.31 ± 5.38	18.01 ± 4.13
Adult	19.16 ± 1.78	0.51 ± 0.06	21.77 ± 1.72	58.96 ± 2.07	18.75 ± 2.76

The ultrastructural development and 3D reconstruction of the transparent carapace of the ostracod *Skogsbergia lerneri*

Benjamin M. Rumney[1] (0000-0001-7854-9739), Farhana T. Malik[2] (0000-0003-4315-5726), Siân R. Morgan[1] (0000-0003-4322-5763), Andrew R. Parker[3] (0000-0002-4564-2838), Simon Holden[4], Julie Albon[1] (0000-0002-3029-8245), Philip N. Lewis[1] (0000-0003-4253-998X) and Keith M Meek[1] (0000-0002-9948-7538)

[1] School of Optometry and Vision Sciences, Cardiff University, Maindy Road, Cardiff, UK

[2] Swansea University, School of Management, Swansea, SA1 8EN,

[3] Green, Templeton College, University of Oxford, Woodstock Road, Oxford, OX2 0HG, UK,

[4] DSTL Physical Sciences Group, Platform Systems Division, DSTL Porton Down, Salisbury, UK

Corresponding author: Philip N. Lewis, Email: lewispn@cardiff.ac.uk
