# Supplementary material for: The ultrastructural development and 3D reconstruction of the transparent carapace of the ostracod Skogsbergia lerneri
Source: Mar Biol. 2022 Feb 13;169(3):35. doi: 10.1007/s00227-021-04006-7 (PMC8841342; doi:10.1007/s00227-021-04006-7)
Supplement: Supplementary file 5 — Supplementary file5 (PDF 129 KB) [file 227_2021_4006_MOESM5_ESM.pdf]

Online Resource 5 The thickness of each layer of the carapace throughout development (number of animals used, n = 5 for each instar)

Instar	Epicuticle Thickness (nm)	Exocuticle Thickness (μm)	Endocuticle Thickness (μm)	Membranous layer Thickness (μm)
1	50.03 ± 4.14	0.47 ± 0.10	0.37 ± 0.10	0.64 ± 0.24
2	79.22 ± 8.434	1.44 ± 0.09	3.38 ± 0.84	1.21 ± 0.42
3	100 ± 9.6	1.94 ± 0.41	6.08 ± 1.12	1.67 ± 0.51
4	89.85 ± 30.63	1.37 ± 1.00	2.60 ± 1.71	1.4 ± 1.18
5	95.92 ± 14.01	2.78 ± 0.31	6.31 ± 0.84	1.98 ± 0.50
Adult	97.93 ± 6.83	4.21 ± 0.67	11.34 ± 0.78	3.66 ± 0.91

The ultrastructural development and 3D reconstruction of the transparent carapace of the ostracod *Skogsbergia lerneri*

Benjamin M. Rumney[1] (0000-0001-7854-9739), Farhana T. Malik[2] (0000-0003-4315-5726), Siân R. Morgan[1] (0000-0003-4322-5763), Andrew R. Parker[3] (0000-0002-4564-2838), Simon Holden[4], Julie Albon[1] (0000-0002-3029-8245), Philip N. Lewis[1] (0000-0003-4253-998X) and Keith M Meek[1] (0000-0002-9948-7538)

[1] School of Optometry and Vision Sciences, Cardiff University, Maindy Road, Cardiff, UK

[2] Swansea University, School of Management, Swansea, SA1 8EN,

[3] Green, Templeton College, University of Oxford, Woodstock Road, Oxford, OX2 0HG, UK,

[4] DSTL Physical Sciences Group, Platform Systems Division, DSTL Porton Down, Salisbury, UK

Corresponding author: Philip N. Lewis, Email: lewispn@cardiff.ac.uk
