# Supplementary material for: The ultrastructural development and 3D reconstruction of the transparent carapace of the ostracod Skogsbergia lerneri
Source: Mar Biol. 2022 Feb 13;169(3):35. doi: 10.1007/s00227-021-04006-7 (PMC8841342; doi:10.1007/s00227-021-04006-7)
Supplement: Supplementary file 6 — Supplementary file6 (PDF 260 KB) [file 227_2021_4006_MOESM6_ESM.pdf]

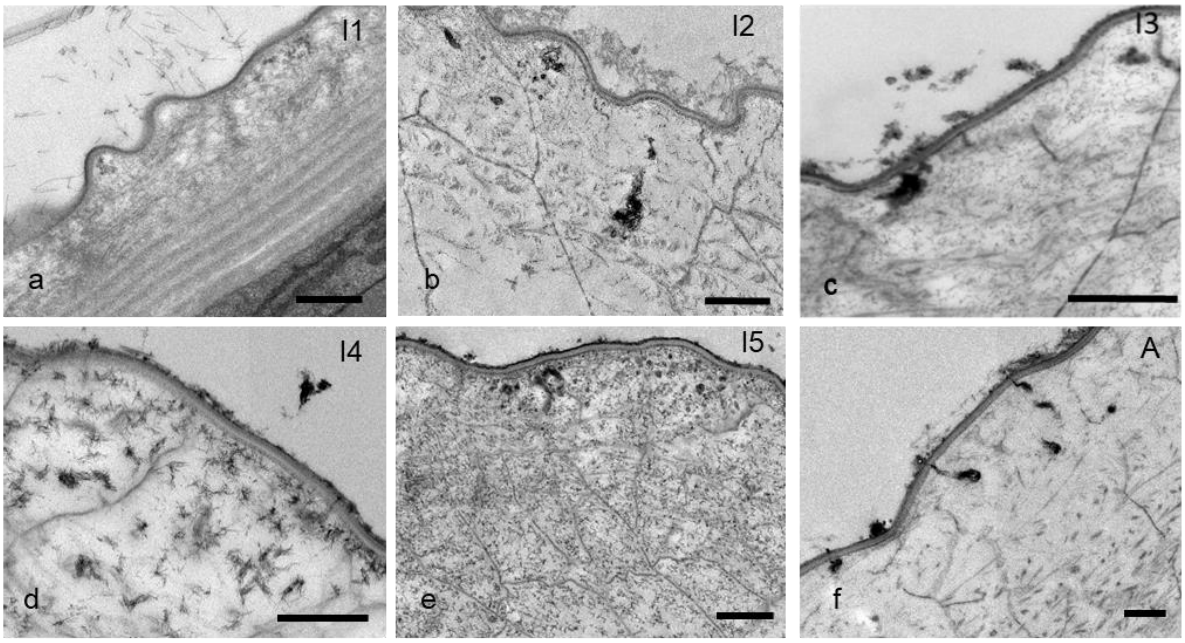

Online Resource 6 TEM images of the epicuticle and exocuticle at each stage of development **a)** instar 1, **b**) instar 2, **c)** instar 3, **d)** instar 4**, e)** instar 5 and **f)** the adult. The chitin within the exocuticle can be seen to follow a specific orientation. Scale bars represent 500 nm

The ultrastructural development and 3D reconstruction of the transparent carapace of the ostracod *Skogsbergia lerneri*

Benjamin M. Rumney[1] (0000-0001-7854-9739), Farhana T. Malik[2] (0000-0003-4315-5726), Siân R. Morgan[1] (0000-0003-4322-5763), Andrew R. Parker[3] (0000-0002-4564-2838), Simon Holden[4], Julie Albon[1] (0000-0002-3029-8245), Philip N. Lewis[1] (0000-0003-4253-998X) and Keith M Meek[1] (0000-0002-9948-7538)

[1] School of Optometry and Vision Sciences, Cardiff University, Maindy Road, Cardiff, UK

[2] Swansea University, School of Management, Swansea, SA1 8EN,

[3] Green, Templeton College, University of Oxford, Woodstock Road, Oxford, OX2 0HG, UK,

[4] DSTL Physical Sciences Group, Platform Systems Division, DSTL Porton Down, Salisbury, UK

Corresponding author: Philip N. Lewis, Email: lewispn@cardiff.ac.uk
