# Supplementary material for: The ultrastructural development and 3D reconstruction of the transparent carapace of the ostracod Skogsbergia lerneri
Source: Mar Biol. 2022 Feb 13;169(3):35. doi: 10.1007/s00227-021-04006-7 (PMC8841342; doi:10.1007/s00227-021-04006-7)
Supplement: Supplementary file 7 — Supplementary file7 (PDF 128 KB) [file 227_2021_4006_MOESM7_ESM.pdf]

Online Resource 7 The height and width of the basal and medial calcified polyhedrons at each of the developmental stages (number of animals used, n = 5 for each instar)

Instar	Basal polyhedron Height (µm)	Basal Polyhedron Width (µm)	Medial Polyhedron Height (µm)	Medial Polyhedron Width (µm)
1	N/A	N/A	N/A	N/A
2	0.46 ± 0.12	0.52 ± 0.11	1.63 ± 0.40	1.4 ± 0.23
3	0.42 ± 0.11	0.54 ± 0.09	1.24 ± 0.49	1.36 ± 0.45
4	0.53 ± 0.23	0.56 ± 0.11	1.52 ± 0.50	1.55 ± 0.47
5	0.77 ± 0.30	0.62 ± 0.09	2.01 ± 0.28	1.67 ± 0.36
Adult	0.34 ± 0.14	0.39 ± 0.13	1.07 ± 0.31	1.02 ± 0.38

The ultrastructural development and 3D reconstruction of the transparent carapace of the ostracod *Skogsbergia lerneri*

Benjamin M. Rumney[1] (0000-0001-7854-9739), Farhana T. Malik[2] (0000-0003-4315-5726), Siân R. Morgan[1] (0000-0003-4322-5763), Andrew R. Parker[3] (0000-0002-4564-2838), Simon Holden[4], Julie Albon[1] (0000-0002-3029-8245), Philip N. Lewis[1] (0000-0003-4253-998X) and Keith M Meek[1] (0000-0002-9948-7538)

[1] School of Optometry and Vision Sciences, Cardiff University, Maindy Road, Cardiff, UK

[2] Swansea University, School of Management, Swansea, SA1 8EN,

[3] Green, Templeton College, University of Oxford, Woodstock Road, Oxford, OX2 0HG, UK,

[4] DSTL Physical Sciences Group, Platform Systems Division, DSTL Porton Down, Salisbury, UK

Corresponding author: Philip N. Lewis, Email: lewispn@cardiff.ac.uk
