# Supplementary material for: The ultrastructural development and 3D reconstruction of the transparent carapace of the ostracod Skogsbergia lerneri
Source: Mar Biol. 2022 Feb 13;169(3):35. doi: 10.1007/s00227-021-04006-7 (PMC8841342; doi:10.1007/s00227-021-04006-7)
Supplement: Supplementary file 9 — Supplementary file9 (PDF 127 KB) [file 227_2021_4006_MOESM9_ESM.pdf]

Online Resource 9 The number of membranous layer lamellae at each developmental stage and their corresponding changes in both their thickness and the spacing between them (number of animals used, n = 5 for each instar)

Instar	Mean number of membranous layer lamellae	Mean lamellar thickness (nm)	Mean inter-lamellar space (nm)
1	5.4 ± 1.8	67.9 ± 9.8	42.4 ± 4.1
2	7.7 ± 1.6	98.1 ± 10.2	59.0 ± 19.4
3	8.8 ± 0.9	58.8 ± 11.0	33.2 ± 8.6
4	10.0 ± 1.1	71.2 ± 21.1	51.0 ± 17.4
5	10.0 ± 2.0	95.9 ± 15.2	73.4 ± 14.3
Adult	17.6 ± 4.6	75.9 ± 13.7	44.1 ± 4.4

The ultrastructural development and 3D reconstruction of the transparent carapace of the ostracod *Skogsbergia lerneri*

Benjamin M. Rumney[1] (0000-0001-7854-9739), Farhana T. Malik[2] (0000-0003-4315-5726), Siân R. Morgan[1] (0000-0003-4322-5763), Andrew R. Parker[3] (0000-0002-4564-2838), Simon Holden[4], Julie Albon[1] (0000-0002-3029-8245), Philip N. Lewis[1] (0000-0003-4253-998X) and Keith M Meek[1] (0000-0002-9948-7538)

[1] School of Optometry and Vision Sciences, Cardiff University, Maindy Road, Cardiff, UK

[2] Swansea University, School of Management, Swansea, SA1 8EN,

[3] Green, Templeton College, University of Oxford, Woodstock Road, Oxford, OX2 0HG, UK,

[4] DSTL Physical Sciences Group, Platform Systems Division, DSTL Porton Down, Salisbury, UK

Corresponding author: Philip N. Lewis, Email: lewispn@cardiff.ac.uk
